# Efficacy of Expired Antibiotics: A Real Debate in the Context of Repeated Drug Shortages

**DOI:** 10.3390/antibiotics13050466

**Published:** 2024-05-19

**Authors:** Benjamin Davido, Hugues Michelon, Christel Mamona, Pierre de Truchis, Karim Jaffal, Azzam Saleh-Mghir

**Affiliations:** 1 Hôpital Raymond Poincaré, Université Paris-Saclay, 92380 Garches, France; hugues.michelon@aphp.fr (H.M.); christel.mamonakilu@aphp.fr (C.M.); p.de-truchis@aphp.fr (P.d.T.); 2 UMR 1173, Infection et Inflammation, Université Versailles-St-Quentin, 78000 Versailles, France; azzam.saleh-mghir-ext@aphp.fr; 3 Hôpital Ambroise Paré, Université Paris-Saclay, 92100 Boulogne-Billancourt, France; karim.jaffal@aphp.fr

**Keywords:** expired drugs, expiry date, antibiotic shortages

## Abstract

This narrative review aims to discuss the main interest in and cautions associated with the use of expired antibiotics in the context of repeated shortages, notably in Europe. Articles concerning the topic of expiry dates related to antibiotic use were reviewed using keywords in the PubMed^®^/MEDLINE and Google Scholar databases to identify the most extensive evidence-based documentation. The present review evaluates the potential interest and efficacy of using expired drugs and their possible related adverse events. Overall, in the context of drug shortages, expiry dates could be safely extended for at least one year for most solid antibiotics (tablets or powder) used in daily clinical practice, as long as they are stored under the right conditions, in accordance with the summary of product characteristics.

## 1. Introduction

In the context of the worldwide increased prevalence of multidrug-resistant organisms (MDROs), it is widely acknowledged that antibiotic stewardship programs are necessary to tackle antimicrobial resistance. On the one hand, the burden of antimicrobial resistance is concerning in treating childhood infections in large parts of South Asia or the Pacific, with antibiotics no longer being effective [1], which is detrimental. Furthermore, it has been established that antimicrobial resistance was responsible for approximately 35,000 deaths in Europe in 2015 [2], which advocates promoting the appropriate use of antibiotics. On the other hand, few studies have focused on recurrent antibiotic shortages. A recent example is the drug shortage of cefazolin in Japan, which led to inappropriate antibiotic use and confusion among prescribers [3,4]. Likewise, the shortage of cefepime in Switzerland was responsible for the increased consumption of piperacillin-tazobactam and carbapenems, and ultimately, responsible for a decrease in the susceptibility of *P. aeruginosa* in hospitals [5].

The COVID-19 pandemic exacerbated medicine shortages, partly due to disruptions in supply chains or the manufacturing of active pharmaceutical ingredients and discontinuations in drug marketing. For instance, in late 2022, a worldwide alert about penicillin shortages [6] coincided with an increased prevalence of group A streptococcal infections among children in Europe [7] which has been associated with a serious global economic burden [8].

In addition to minimizing medication wastage, some countries, such as the United States have implemented unit dose dispensing since the 1960s to deliver drugs, including antibiotics, providing the exact number of tablets required based on the prescribed dose and duration [9]. Furthermore, this increased prevalence of MDROs will inevitably lead to an increase in antibiotic consumption and calls for rethinking a strategy for the sustainable use of antibiotics. Altogether, these observations prompt us to reconsider strategies for mitigating antibiotic shortages and optimizing the use of available antibiotics up to their expiration dates and beyond. Here, we reviewed the literature regarding the potential benefits of using antibiotics beyond their expiry dates and also evaluated the risks of adverse events.

## 2. Materials and Methods

This narrative review of the literature focused on the studies related to the use of antibiotics beyond the expiry date reported in the PubMed and Google Scholar databases through March 2024. Two reviewers conducted the research: one infectious disease specialist and one research engineer. The keywords used were “outdated drugs”, “antibiotic shortages”, “antibiotic expiry date”, and “drugs expiration date”, in all fields. Original articles, reviews, and viewpoints were all considered. Both reviewers independently screened the titles and abstracts of all retrieved articles. Only English-language articles were included after selecting those that were deemed suitable for full-text screening. This decision was primarily motivated by ethical considerations, as the few foreign articles available were letters that mostly reproduced the English literature verbatim, constituting potential plagiarism.

## 3. Results

The initial search of the scientific literature yielded more than 1000 articles, including those describing all types of drugs related to drug expiration dates. After being independently screened by two reviewers, 29 articles were selected for full-text screening. Based on this screening, 12 publications were withdrawn due to their limited contribution, including duplicates (as mentioned above); therefore, a total of 17 articles focusing on antibiotics were deemed suitable for the review.

### 3.1. Using Antibiotics beyond the Expiry Date

Sandford-Smith supported in 2003 that outdated drugs, in general, may still be useful, responding to a colleague in Nepal who advocated for discarding expired drugs, including antibiotics [10].

It is important to note that the use of expired drugs has been established by the US Department of Defense (DoD) (Arlington, VA, USA) through the FDA Shelf-Life Extension Program (SLEP) since 1986 [11] and by the German armed forces (Bundeswehr), to look into deferring the replacement cost of expiring DoD stockpiles. Indeed, postponing the replacement of expired drugs might save about $200.000 per year according to a report by the Mayo Clinic [12]. This money could potentially be reinvested in drug research and development with the support of stakeholders.

#### 3.1.1. Stability Profile and Long-Term Storage

To obtain marketing authorization, the manufacturer must provide data on the stability profiles of the drug for a defined expiration date with specific storage conditions to ensure the closed container remains stable at the expiry date. Most products have been labeled for durations ranging from 1 to 5 years [13].

Data from the DoD SLEP reported the stability of several drugs past their expiration dates and have shown that 2650 of 3005 lots (−88%) corresponding to 122 different products stored in their unopened original containers remained stable for an average of 66 months after their expiration dates [14]. Remarkably, none of them failed within 1 year, and about 12% of the lots remained stable for more than 4 years after the expiration date.

Heat and humidity can play a role in the degradation of some drug formulations, but Stark et al. reported that cefoxitin sodium powder stored under heavy conditions (40 °C, 75% humidity) remained effective after more than 1.5 years [15].

Humidity brings moisture and H_2_O ions, which can destabilize the chemical properties of drugs, such as the beta-lactam core, leading to antibiotic expiration and reduced effectiveness. Moreover, expired drugs, including antibiotics, have been suggested as corrosion inhibitors, potentially decreasing the corrosion rate of metals and alloys, thus highlighting the significance of the biological and chemical properties of drugs [16].

#### 3.1.2. Drug Formulations

Solutions and suspensions are generally less stable than solid dosage forms (tablets, capsules, or powders). An indication of potential drug expiry is the discolored aspect of the liquid or signs of precipitation with crystals or freezing in solution, particularly for injectables. In such cases, the drug should not be used. Conversely, storing the drug in a cool place, such as a refrigerator, will maintain its efficacy for many years. It has even been described that antibiotics remained effective for several decades after their manufacture when tested from pure drug substances [17].

For clarification, we summarized all antibiotics in Table 1, classified according to dosage form, number of lots tested, extension time in months, and other parameters if applicable. Overall, we gathered 758 lots, with a mean extension period of 120 months (ranging from 4–564).

### 3.2. Avoiding Using Antibiotics beyond the Expiry Date

Considering the specificity of drug formulations, the use of expired oral antibiotics in pediatrics is not recommended. Indeed, Ogunshe et al. showed in their in vitro study that, apart from reduced efficacy, the administration of expired antibiotics can lead to increased antibiotic resistance, clinical treatment failure, and possible adverse drug reactions [19]. Of note, this study utilized antibiotics in syrup form, as commonly used in pediatrics. Typically, drugs in solution, such as drops or syrups, have relatively short expiry dates after opening, ranging from 2 weeks to 2 months, and must be stored in a refrigerator [20].

In addition, while it has been suggested that unused drugs may remain effective for more than 90% of lots for up to at least 5 years [13], it is important to note that the remaining 10% may be ineffective, posing a significant risk of unfavorable outcomes, especially for critical infections.

Another study evaluated the efficacy of expired and active forms of various antibiotics, including amoxicillin, cefixime, and sparfloxacin (a fluoroquinolone), against Saccharomyces cerevisiae. The study revealed that for low concentrations of antibiotics, typically less than 10 mg/mL, the optical densities tabulated for microbial growth were significantly higher when using expired drugs. The authors concluded that expired forms of antibiotics had drastically lost their efficacy [21].

Regarding safety concerns, a small number of cases have been reported involving the expired oral antibiotic tetracycline, resulting in toxic reactions, including kidney failure, specifically Fanconi syndrome [22,23]. Many attribute this toxicity to degradation products of tetracycline (such as epi-anhydrotetracycline or anhydrotetracycline). Patients experienced symptoms such as nausea, vomiting, and metabolic acidosis within 2 to 8 days of taking the expired antibiotic. However, no recent cases of toxicity related to expired oral antibiotic tetracycline or its derivatives, such as doxycycline, have been reported in recent years.

### 3.3. Potential Benefits of Using Expired Drugs

One risk of discarding antibiotics while they are still effective is the environmental impact. Studies have shown that the presence of antibiotics in water bodies may contribute to antibiotic resistance [24,25]. By extending the life cycle of antibiotics, it may limit the cycle of antibiotic waste, and therefore, reduce the subsequent development of antibiotic resistance.

Another concern is the ethical dilemma of discarding drugs when they are needed, particularly during outbreaks when there is an urgent requirement for antivirals or antibiotics [26]. For example, this issue was raised during the H1N1 pandemic when there was a shortage of oseltamivir in 2009 and more recently during the COVID-19 pandemic. This was addressed by the FDA, which extended the expiry dates of this antiviral for 10 years [27]. These decisions suggest that it could be possible to extend the expiry dates of many anti-infective agents without compromising patient safety.

Moreover, discarding expired drugs has several drawbacks. For example, many low- or middle-income countries cannot afford to replenish their pharmaceutical supplies regularly, leading to difficulties in maintaining adequate stockpiles of essential antibiotics to combat infections. Some authors have even suggested redirecting expired medications that have been deemed safe for use to these countries, thereby preventing drug wastage and conserving resources for other healthcare initiatives [28,29].

## 4. Discussion

After more than 50 years, the debate over the use of expired medication remains contentious. The literature suggests that the use of expired antibiotics may be considered after thorough patient education. This is supported by findings indicating that no antibiotic tested failed to be effective after one year. Additionally, a significant portion of pharmaceutical stability data relies on imperfect linear and exponential models for estimating drug expiry [30]. Moreover, pharmaceutical manufacturers are required to assign an expiration date to each marketed drug product. The labeled shelf life is estimated using appropriate stability testing International Conference on Harmonization (ICH) Q1A (R2). The stability assessments of both the new drug substance and the new drug product stored under controlled conditions are required documentation for submission to regulatory authorities. The initial expiration date is then based on the amount of real-time stability data (generally from pilot-scale batches) available at the time of the approval of the drug. This initial date may subsequently be extended subject to the continuation of stability studies provided at the discretion of the manufacturer.

One crucial factor to consider is the current shortage situation of the selected antibiotic, which may influence this decision. It appears reasonable to suggest that for tablets and other solid antibiotics, extending the life cycle by using antibiotics beyond a one-year expiry period, when necessary, particularly in the event of recurrent shortages or in countries lacking access to renew their strategic stockpile. Moreover, prior to the 1999 World Health Organization (WHO) (Geneva, Switzerland) drug donation guidelines, a study found that the median expiration times when shipments were made by the organization were 599 and 550 days; about 30% of items shipped had a shelf life of one year or less, and about 6% had a shelf life of less than 100 days [31]. This highlights the complexity of ensuring proper and optimal utilization of medications even after 1999, according to a literature review [32].

One key aspect when prescribing antibiotics is adhering to antimicrobial stewardship principles that can be challenged in cases of drug shortage. Indeed, antimicrobial stewardship relies schematically on three principles: a target therapy with appropriate indication, the right dose, and duration of treatment [33]. An example of a situation to avoid is the use of broad-spectrum beta-lactams with clindamycin during the supply disruption of cefazolin in Japan [4]. This illustrates the unintended consequences of the lack of proactive action and framework provided by health authorities regarding extending drug expiration dates during unexpected crises such as shortages.

The issue of expired medications also raises the absence of particular legislation to control the disposal of expired medication. Currently, in the era of increased prevalence of MDROs and recurrent antibiotic shortages, adopting particular legislation to control the disposal of expired medication is an urgent need. Innovative stability tests would help in reducing the annual volume of expired medications. Such an approach could be a new revolution in the field of pharmacy by creating an opportunity for new recycling companies that are different from synthetic companies. Their main duties would be extracting, purifying, and repacking. Although such programs could be losing out in economic burden, it would be an environmentally beneficial strategy.

Furthermore, the instructions for proper disposal practices on the package, for instance “please contact your local drug take-back program for the disposal of this product”, would be greatly contribute to raising public awareness regarding the appropriate disposal practices. Indeed, the accumulation of pharmaceutical waste imposes ecological, economic, and social/ethical burdens. Unfortunately, medication wastage cannot be attributed solely to drug expiry but also to several other factors, including prescription-only medicines, non-expired medicines, and medicines for chronic diseases. In a study conducted in Austria in 2018, it was estimated that, extrapolated for Vienna, at least €37.65 million were wasted in household garbage, corresponding to €21 per inhabitant, reminding us of the potential cost savings that could be achieved every year [34].

The major drawback of advocating for the use of expired antibiotics is the real risk of seeing pharmaceutical companies increase the price of their products due to the lack of profits, which could be detrimental to research and development activities. Also, it should be noted that in most countries, regulations and legislation discourage pharmacists and healthcare providers from dispensing outdated drugs [24]. As recently as 2019 in the USA, one pharmacy group branded CVS was blamed by the state board of pharmacy for dispensing expired medication to a patient. Furthermore, it has been reported in the press that 142 CVS stores in over 41 counties sold expired products, representing up to 60 percent of the stores. Some of the items were more than two years past their expiration dates [35].

Furthermore, for more effective prescribing of medications, including antibiotics, it would be beneficial for most countries to adopt the American model of unit dose drug distributions in primary care to minimize wastage and potentially reduce costs. Similarly, in France, since 2017, the principle of a medication co-payment of 50 cents per box consumed has been introduced to encourage patient responsibility.

## 5. Conclusions

Overall, it would be more relevant to establish a theoretical expiry date after which the effectiveness cannot be guaranteed to be 100%, especially in cases of inadequate storage conditions, rather than formally limiting the prescription. Indeed, considering the literature, most antibiotics’ expiry dates can be safely extended after one year and may benefit patients, particularly in conditions of shortage in low- and middle-income countries with difficulties accessing stockpiles. After one year and up to five years, consideration should be given to the availability of alternative medications while adhering to the principles of antimicrobial stewardship.

## Figures and Tables

**Table 1:** Antibiotic efficacy tested in the literature according to extension times.

Antibiotic (INN)	Dosage Form	Number of Lots	Mean Extension Time Period (Months)	Efficacy (Content of the Active Substance) %	Mean Time Period (Months) until Active Substance Degradation	Reference
Amoxicillin	tablets	21	23	100	-	Lyon et al. [14]
Ampicillin	capsules	5	49	100	-	Lyon et al. [14]
Ampicillin	injection-solution	8	57	87.5	42	Lyon et al. [14]
Ampicillin	pure drug substance *	1	252	100	-	Zilker et al. [17]
Cefazolin	powder	10	82	80	82	Lyon et al. [14]
Cefoxitin	powder	10	24	60	24	Lyon et al. [14]
Cefoxitin	powder	1	70	100	-	Stark et al. [15]
Ceftriaxone	powder	4	60	100	-	Lyon et al. [14]
Cephalexin	capsules	6	57	100	-	Lyon et al. [14]
Cephapirin sodium	powder	13	74	92.3	98	Lyon et al. [14]
Ciprofloxacin	tablets	242	55	100	-	Lyon et al. [14]
Ciprofloxacin	suspension	7	32	100	-	Lyon et al. [14]
Ciprofloxacin	pure drug substance *	1	300	100	-	Zilker et al. [17]
Clindamycin	injection-solution	31	44	80.6	44	Lyon et al. [14]
Colistin	pure drug substance *	1	492	100	-	German et al. [18]
Doxycycline	pure drug substance *	1	432	100	-	German et al. [18]
Doxycycline	capsules	13	50	100	-	Lyon et al. [14]
Doxycycline	powder	31	27	100	-	Lyon et al. [14]
Doxycycline	tablets	169	27	98.2	27	Lyon et al. [14]
Erythromycin	powder	60	4	100	-	Lyon et al. [14]
Flucloxacin	capsule	1	50	100	-	Stark et al. [15]
Ofloxacin	pure drug substance *	2	264	100	-	Zilker et al. [17]
Oxacillin	powder	13	56	100	-	Lyon et al. [14]
Neomycin	ophthalmic ointment	5	28	80	28	Lyon et al. [14]
Oxytetracycline	pure drug substance *	3	504	100	-	German et al. [18]
Penicillin G benzathine	suspension	4	70	100	-	Lyon et al. [14]
Penicillin G	powder	15	49	93.3	49	Lyon et al. [14]
Penicillin G	powder	7	70	28.6	70	Lyon et al. [14]
Spectinomycin	suspension	8	83	87.5	83	Lyon et al. [14]
Spiramycin	pure drug substance *	1	564	100	-	German et al. [18]
Sulfacetamide	ophthalmic ointment	4	39	75	39	Lyon et al. [14]
Sulfadiazine	cream	37	57	97.3	53	Lyon et al. [14]
Sulfadoxine pyrimethamine	tablets	8	67	87.5	58	Lyon et al. [14]
Sulfisoxazole	tablets	4	56	100	-	Lyon et al. [14]
Tetracycline	capsules	11	50	63.6	32	Lyon et al. [14]

* For pure drug substances, the extension time has been considered based on the age of the product, as it was not commercialized.
